# Influence of Body Composition and Specific Anthropometric Parameters on SIBO Type

**DOI:** 10.3390/nu15184035

**Published:** 2023-09-18

**Authors:** Justyna Paulina Wielgosz-Grochowska, Nicole Domanski, Małgorzata Ewa Drywień

**Affiliations:** 1 Department of Human Nutrition, Institute of Human Nutrition Sciences, Warsaw University of Life Sciences, 02-776 Warsaw, Poland; justyna_wielgosz@sggw.edu.pl; 2 Faculty of Pharmaceutical Sciences, University of British Columbia, Vancouver, BC V6T 1Z3, Canada; nicole.domanski@ubc.ca

**Keywords:** SIBO, gut, hydrogen–methane, dysbiosis, BMI, body fat, body composition, breath test

## Abstract

Recent observations have shown that Small Intestinal Bacterial Overgrowth (SIBO)affects the host through various mechanisms. While both weight loss and obesity have been reported in the SIBO population due to alterations in the gut microbiome, very little is known about the influence of SIBO type on body composition. This study aimed to evaluate whether there is a link between the three types of SIBO: methane dominant (M+), hydrogen dominant (H+), and methane–hydrogen dominant (H+/M+) and specific anthropometric parameters. This observational study included 67 participants (W = 53, M = 14) with gastrointestinal symptoms and SIBO confirmed by lactulose hydrogen–methane breath tests (LHMBTs) using the QuinTron device. Participants underwent a body composition assessment by Bioelectrical Impedance Analysis (BIA) using the InBody Analyzer. In the H+/M+ group, body weight (*p* = 0.010), BMI (*p* = 0.001), body fat in kg (*p* = 0.009), body fat in % (*p* = 0.040), visceral fat (*p* = 0.002), and mineral bone content (*p* = 0.049) showed an inverse correlation with hydrogen (H_2_) gas production. These findings suggest that body weight, BMI, body fat, and mineral bone content may be inversely linked to the production of hydrogen and the risk of hydrogen–methane SIBO.

## 1. Introduction

In a state of eubiosis within the gut microbiota, the human body relies on a diverse array of host defense mechanisms, such as gastric acid, myoelectric complexes (MMCs), the ileophilic valve, intrasecretory immunoglobulin A, and pancreatic enzymes to effectively counteract excessive microorganism overgrowth [1]. The deregulation of any of these protective mechanisms can lead to microbiota dysbiosis, a potential pathway for the development of Small Intestinal Bacterial Overgrowth (SIBO) [2,3]. SIBO is characterized by increased colonization of anaerobic and aerobic microorganisms within the small intestine, predominantly Gram-negative species including *Klebsiella pneumoniae, Escherichia coli*, *Streptococcus gramineus*, *Prevotella*, *Clostridium* spp, and *Methanobrevibactersmithii* [4,5,6]. Depending on the dominant composition of the bacterial overgrowth, the production of gases as a result of bacterial fermentation, such as hydrogen (H_2_), methane (CH_4_), or hydrogen sulfide (H_2_S) is observed. On this basis, four types of SIBO (hydrogen dominant, methane dominant, hydrogen–methane dominant, and hydrogen–sulfide dominant) have been distinguished [7,8,9]. Hydrogen-dominant SIBO (H+) is often associated with the diarrhea subtype of IBS, whereas methane-dominant SIBO (M+) mainly manifests as constipation. Hydrogen–sulfide-dominant SIBO (S+) is characterized by intense gas production, while hydrogen–methane-dominant SIBO (H+/M+) presents with diverse symptoms ranging from abdominal pain, reflux, and stomach discomfort to fatigue [10,11,12,13,14]. The prevalence of SIBO is not precisely defined, but it is estimated to range from 2.5% to 22% in adults [3]. The frequency of SIBO occurrence in children is challenging to determine due to limited studies.

Managing SIBO and addressing the decreased quality of life in affected patients necessitates collaboration between multiple specialists [15]. Recently, non-invasive and cost-effective breath tests have emerged as a reliable diagnostic method for SIBO and are now extensively used. These tests offer an alternative to invasive procedures, which were traditionally considered the gold standard for SIBO diagnosis [1,16,17,18]. It is important to note that M+ SIBO may affect up to 30% of the general population, primarily due to colonization by a hydrogen cross-feeder *Methanobrevibacter smithii* which converts H_2_, and carbon dioxide (CO_2_) into CH_4_. Thus, in many methane producers, H+ might not be discovered [8]. Due to this fact, it is crucial to screen patients via hydrogen–methane breath tests [19].

SIBO as a pathology of gut microbiota is often a neglected disorder due to symptomology that overlaps with many other gastrointestinal (GI) diseases. Paradoxically, SIBO can potentially mask an underlying disease, leading to delayed diagnosis, treatment, and exacerbation of the patient’s condition [20,21,22]. The incomplete absorption of carbohydrates in the small intestine, resulting from extensive bacterial fermentation, leads to various mechanisms by which SIBO affects the host. Initially, it interferes with the normal functioning of the digestive tract, giving rise to GI discomfort and ultimately impacting the overall functioning of the entire organism [23]. Additionally, microorganisms compete with the host for micronutrients and macronutrients, causing a lower nutritional status [24,25]. In SIBO patients under the age of 19 years, lower height and body weight were observed based on the height/weight-for-age Z score [26]. Conversely, there have been multiple associations between SIBO and obesity, with the risk of SIBO being up to two times higher in obese patients compared to individuals with a normal BMI [27]. In another study, the frequency of SIBO was 89% in obese people vs. 42.9% in the control group [28]. Being overweight has been linked to alterations in the composition of the gut microbiota, characterized by an increase in methanogenic *Archaea (Methanobrevibactersmithii*) and a disrupted *Bacteroidetes/Firmicutes* ratio, as compared to healthy subjects [29]. Obesity can contribute to increased intestinal permeability, promoting the occurrence of intestinal bacterial overgrowth [30]. Interestingly, bacterial eradication using rifaximin in obese individuals with SIBO did not result in clinically significant changes in body mass [31]. Although weight loss as well as obesity have been reported in the SIBO population, very little is known about the influence of SIBO type on body composition. This study aimed to evaluate whether there is a link between the three types of SIBO: methane-dominant M+, hydrogen-dominant H+, and methane–hydrogen-dominant H+/M+, and body composition and specific anthropometric parameters.

## 2. Materials and Methods

The study was carried out at the Warsaw University of Life Science between September 2021 and January 2022. The inclusion criteria were for adult participants aged 18 to 65 years old and required the presence of abdominal symptoms occurring at least three times per month in the past six months. These participants were recruited from a dietary counseling and medical center who had received a diagnosis of SIBO based on a positive lactulose hydrogen–methane breath test (LHMB). Only individuals who had properly prepared for the breath test were considered eligible for participation in the study. Patients with a history of eating disorders, a diagnosis of celiac disease, IBD, hypoglycemia, and those unable to undergo bioimpedance anthropometric measurements (such as pregnant women or individuals with cardiac pacing/defibrillation devices) were excluded from the study, as these factors constituted key elements required for data collection. A symptom questionnaire was administered to discern each patient’s clinical picture. The study protocol received approval from the Ethics Committee at the Institute of Human Nutrition (Warsaw, Poland), and informed consent was obtained from all subjects prior to their participation in the study.

### 2.1. Anthropometric Data

Participants who fulfilled the study’s eligibility criteria underwent a body composition assessment by Bioelectrical Impedance Analysis (BIA) with the validated InBody 270 Analyzer. The measurements were conducted under the guidance of a registered dietitian. Prior to the test, participants were instructed to refrain from (1) eating 2 h and drinking 1 h prior to test and (2) physical exercise on the day of the assessment. Various parameters related to body composition were evaluated based on electrical conductance. These included body weight (kg), body fat (kg and %), lean body mass (LBM) (kg), skeletal muscle mass (SMM) (kg), visceral fat (cm^2^), waist–hip ratio (WHR), body protein (kg), total body water (L), and bone mineral content (kg). Participant-reported height was recorded. Body mass index (BMI) was calculated as weight divided by height squared (kg/m^2^). BMI categories were classified according to World Health Organization guidelines as “underweight” (BMI < 18.5), “healthy” (18.5 ≤ BMI < 24.9), “overweight” (25 ≤ BMI < 29.9), and “obese” (BMI ≥ 30). Body fat groups were classified as “underfat” (for men <10%; for women <18%), “overfat” (for men >20% for women >28%), and “healthy” (for men 10–20%; for women 18–28%). Visceral fat area was identified as “standard” (VFA < 90 cm^2^), “high” (90 cm^2^ ≤ VFA <140 cm^2)^, or “very high” (VFA ≥ 140 cm^2^) using the InBody scale classifications.

### 2.2. Questionnaire

Patients responded to a comprehensive and validated questionnaire known as the KomPAN^®^ questionnaire, which encompassed demographic information as well as GI complaints such as bloating, diarrhea, constipation, abdominal pain, reflux, and growling. The questionnaire also assessed the frequency of these symptoms.

### 2.3. Lactulose Hydrogen–Methane Breath Test

The QuinTron Instrument Company Data Tracer v 3.0 was utilized to conduct the breath testing, performed by qualified and trained technicians. Participation in the breath test was allowed by meeting the following criteria: (1) no use of antibiotics 4 weeks before the test, (2) no use of probiotics at least 2 weeks before the test, (3) avoiding prokinetics or laxatives 1 week before test, (4) no use of fiber supplementation 3 days preceding the test, (5) adhering to a carbohydrate-restricted diet and consuming only water for 24–48 h prior to the test, (6) fasting for 12 h before the test and during the test day, (7) avoiding smoking and physical activity during the test day. These criteria were in accordance with the North American Breath Testing Consensus Guideline [7]. The outcome of breath test was considered only if the pre-procedure criteria were adhered to by patients. During the breath test, air exhaled by the participants was collected and analyzed for the presence of gases such as H_2_, CH_4_, and CO_2_. The procedure involved obtaining an initial breath sample before administering 10 g of lactulose with 150 mL of water. Then, exhaled gas samples were measured at 20, 40, 80, 90, 100, 120, 140, 160, and 180 min. Subsequently, exhaled gas samples were collected and measured at specific time intervals (20, 40, 80, 90, 100, 120, 140, 160, and 180 min). Throughout the test, participants were allowed to drink up to 500 mL of water. The concentration of hydrogen and methane gases in the breath samples was reported in parts per million (ppm). The detected gases were analyzed and categorized into specific types of SIBO based on predefined guidelines.

### 2.4. Outcome Measures

Using the North American Breath Testing Consensus Guidelines the participants were divided into three groups based on their SIBO subtype (Table 1) [7]. The anthropometric parameters mentioned earlier were compared among these three SIBO groups.

### 2.5. Statistical Analysis

For statistical analysis, the Statistica 13.0 Software was used. A parametric and non-parametric test was used to analyze independent categorical variables. Height, WHR, and total body water were evaluated by Univariate ANOVA, with a post-hoc Tukey test. Age, body fat, lean body mass, skeletal muscle mass, body protein, mineral bone content, visceral fat, BMI, GI symptoms, and mean hydrogen and methane production values were calculated with the use of the non-parametric Kruskal–Wallis test, verified on the basis of the Shapiro–Wilk test, with a post-hoc Bonferroni test. All analyses were considered to be statistically significant with *p* < 0.05. Pearson correlation analyses were used to indicate the correlation between gas production and anthropometric measures in three SIBO types (*p* ≤ 0.05, r ≥ ±0.3).

## 3. Results

### 3.1. Demographics

A total of 67 newly diagnosed SIBO-positive patients, comprising 53 females and 14 males, were included in this study. H+ was observed in 18% (12/67) of adolescents, M+ was identified in 31% (21/67), and H+/M+ was found in 51% (34/67) of the participants. Table 2 provides a comparison of the demographic characteristics and clinical symptoms based on the SIBO subtype. The age distribution was similar across all SIBO groups, with a predominant representation of females in each group. GI symptoms were comparable among the groups, but notable differences were observed in terms of diarrhea and constipation. The H+ group exhibited a higher prevalence of diarrhea compared to the M+ group (*p* = 0.007). Conversely, the M+ demonstrated significantly higher levels of constipation compared to the H+ group (*p* = 0.038). However, the H+/M+ group had similar symptoms according to diarrhea and constipation compared to the other two groups. The H+/M+ group exhibited a tendency towards more pronounced growling (*p* = 0.089), while the M+ group demonstrated the most significant abdominal pain, although it did not reach statistical significance (*p* = 0.242). The frequency of symptoms was similar across the groups, with most patients reporting GI discomfort several times a day.

### 3.2. Hydrogen and Methane Production

Table 3 presents the areas under the curves (AUC) for hydrogen and methane production, as well as the basal concentrations of methane in each SIBO group. The exhaled gases during the lactulose hydrogen–methane breath test (LHMBT) exhibited significant differences among the three SIBO types (*p* = 0.001). The H+ and H+/M+ groups demonstrated higher H_2_ values during the peak time within the first 90 min compared to the M+ group. Moreover, the H+ and H+/M+ groups differed from the M+ group, as it displayed the highest concentration of H_2_ after 90 min of the test and throughout the entire 3h breath testing period (*p* = 0.001). The M+ group was characterized by dominant values of fasting CH_4_ compared to the H+ and H+/M+ groups (*p* = 0.001) and the M+ and H+/M+ groups exhibited a substantial increase in total exhaled CH_4_ levels in comparison to the H+ group.

### 3.3. Body Composition

The characteristics of the selected anthropometric parameters in the study group are presented in Table 4. Height and skeletal muscle mass exhibited statistically significant variations among the different SIBO groups. The H+/M+-dominant patients displayed significantly lower height compared to the H+ group (*p* = 0.023). Likewise, skeletal body mass was found to be the lowest in the H+/M+ group compared to both the M+ and H+ groups (*p* = 0.044). The remaining parameters showed similarities across the three groups; however, there was a trend toward higher body fat percentage (*p* = 0.147) and visceral fat (*p* = 0.170) observed in the H+ group. On the other hand, the M+ group indicated the greatest skeletal muscle mass, water content, mineral bone content, and body protein compared to both the H+ and H+/M+ groups, although these differences did not reach statistical significance.

No statistically significant differences were observed in the percentage of individuals across groups when considering BMI classifications (*p* = 0.991), body fat range (*p* = 0.597), and visceral fat area (*p* = 0.548) (Table 5). However, when examining the percentage of body fat, more than half of the individuals in the H+ and H+/M+ groups indicated excessive adipose tissue (*p* = 0.597), with the H+/M+ group being the only group in which no individuals were classified as having low body fat.

**Figure 1:**
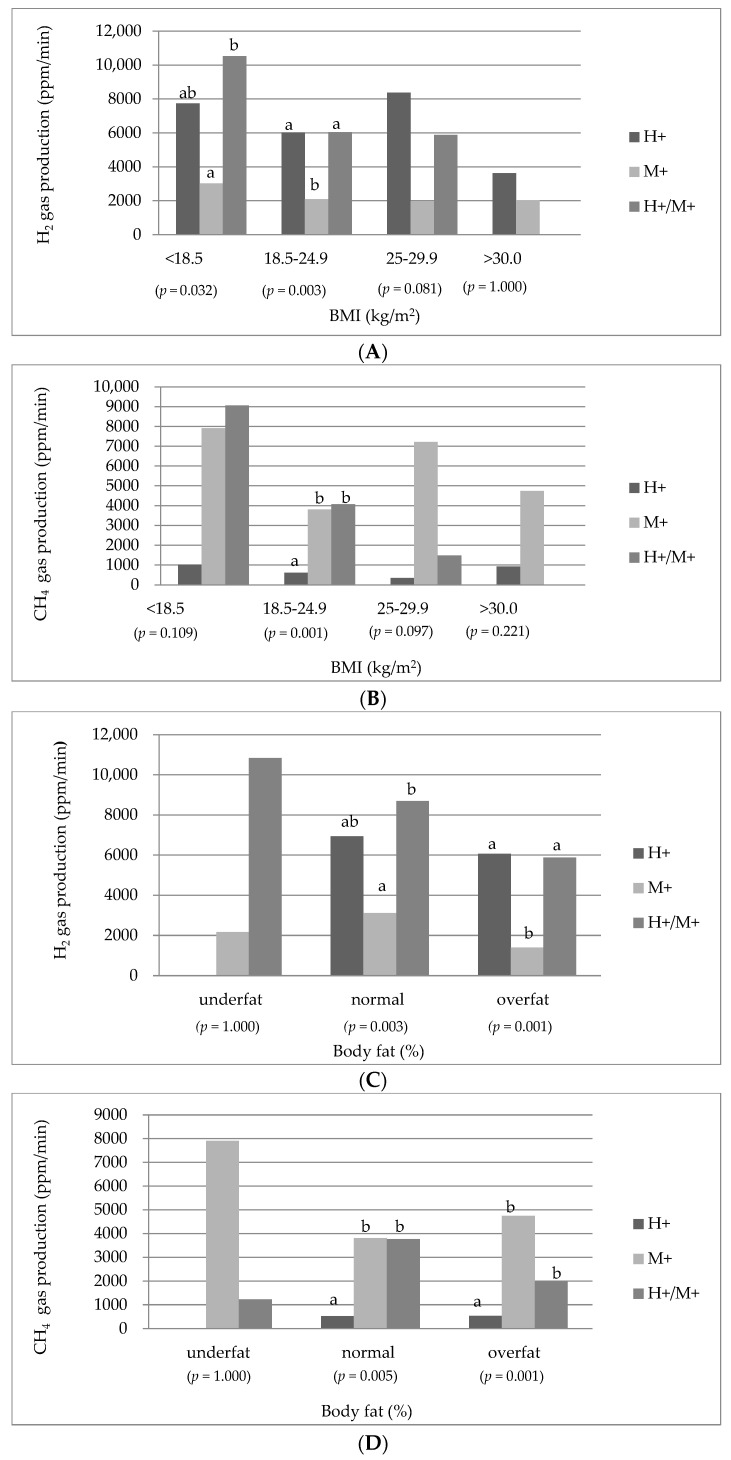

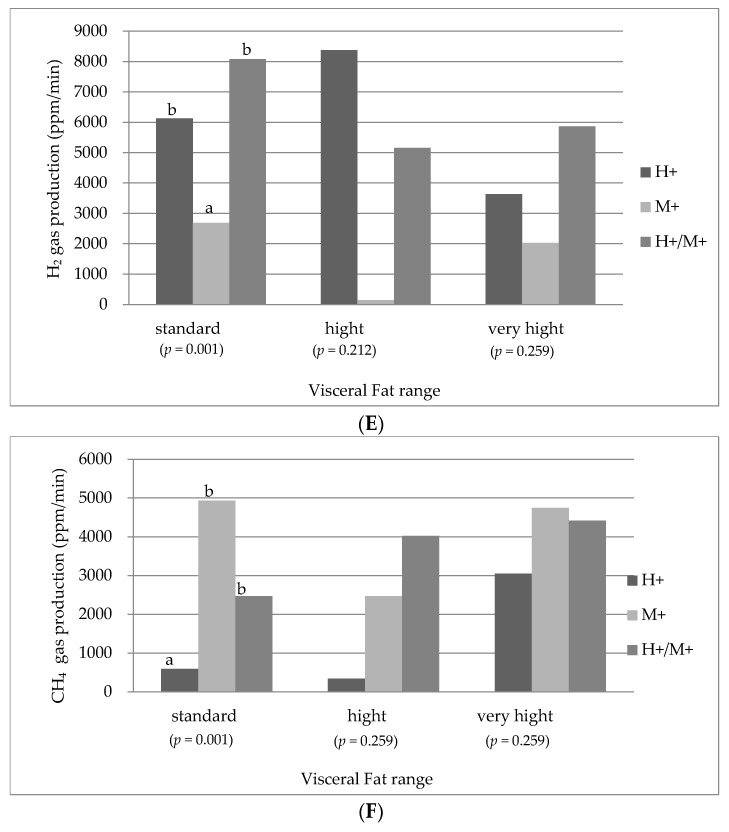
Median H_2_ and CH_4_ concentration as area under the curve (ppm/min) in hydrogen-type, methane-type, hydrogen–methane-type SIBO by BMI classification (**A**,**B**), body fat range (**C**,**D**), visceral fat range (**E**,**F**). *p* value is for comparison of differences among the 3 groups, significance level of *p* = 0.05, values calculated with the use of non-parametric Kruskal–Wallis test, verified on the basis of Shapiro–Wilk test, a-b Bonferroni post-hoc test.

Figure 1 depicts the comparison of the three types of SIBO based on the median production of H_2_ and CH_4_ gases, presented as the area under the curve (ppm/min), during the 180-min breath test following lactulose ingestion, stratified by BMI, body fat range, and visceral fat class. Significant differences were observed in H_2_ production across BMI classes (*p* = 0.032, *p* = 0.003), as well as in CH_4_ production (*p* = 0.001).

According to the classification based on BMI, the production of H_2_ gas tended to be highest in the underweight class within the H+/M+ group, with statistical significance observed when compared to the M+ group (*p* = 0.032). Moreover, the H_2_ production exhibited a progressive decrease across BMI classes in H+/M+ patients. Conversely, within the normal BMI class, the production of H_2_ in the H+ and H+/M+ groups showed statistically comparable values to each other (*p* = 0.003), but differed significantly from the M+ group. Additionally, the levels of exhaled CH_4_ gas in the M+ and H+/M+ groups were proportional to each other but significantly different from the H+ group (*p* = 0.001).

In relation to the body fat range, there were significant differences observed across groups in terms of H_2_ and CH_4_ production, particularly between individuals classified as overfat (*p* = 0.001, *p* = 0.001) and those within the appropriate body fat range (*p* = 0.003, *p* = 0.005). The underfat group showed the highest concentrations of H_2_ and CH_4_, although these differences did not reach statistical significance (*p* = 1.000).

When examining the visceral fat range, significant differences were observed, specifically in the standard category. Patients in the H+ and H+/M+ groups exhibited the highest concentrations of H_2_ (*p* = 0.001), while the M+ and H+/M+ groups displayed the highest CH_4_ production compared to the H+ group (*p* = 0.001).

### 3.4. Inverse Correlations

**Figure 2:**
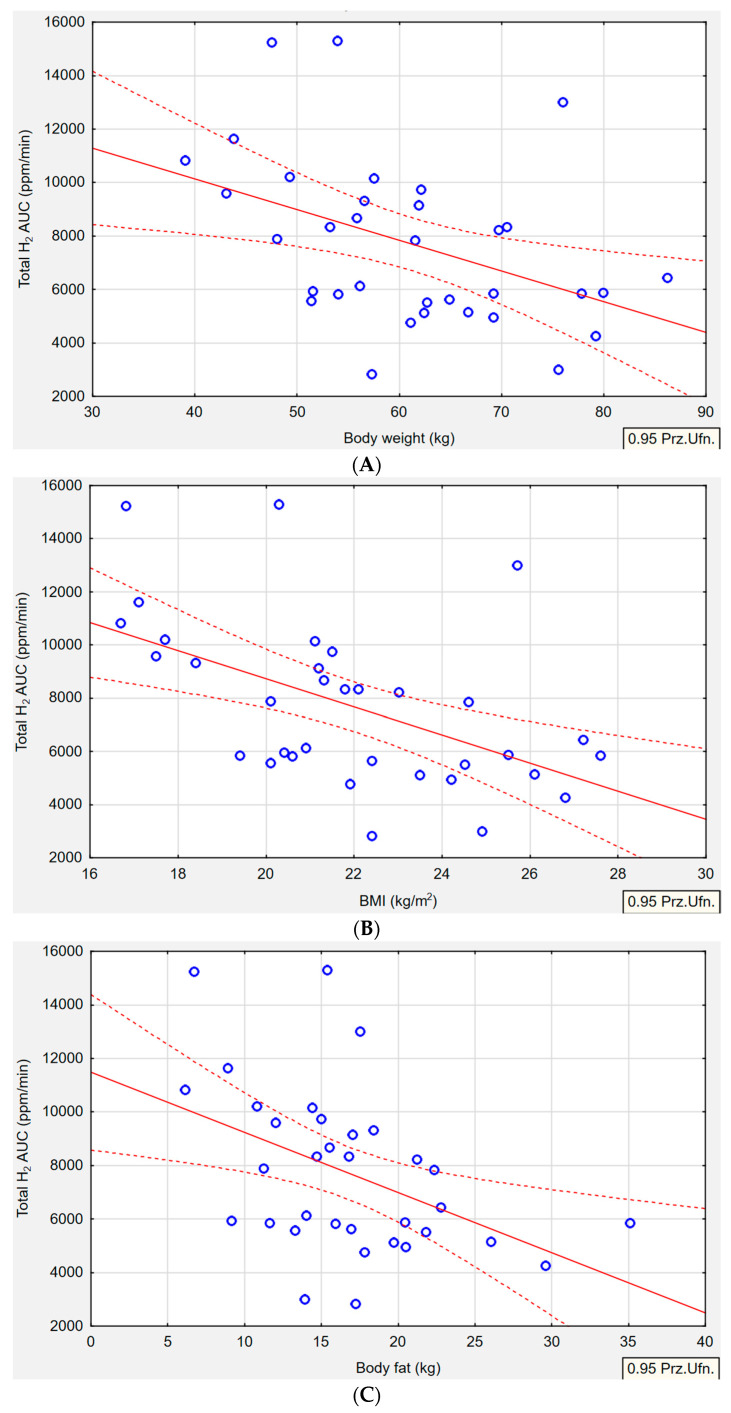

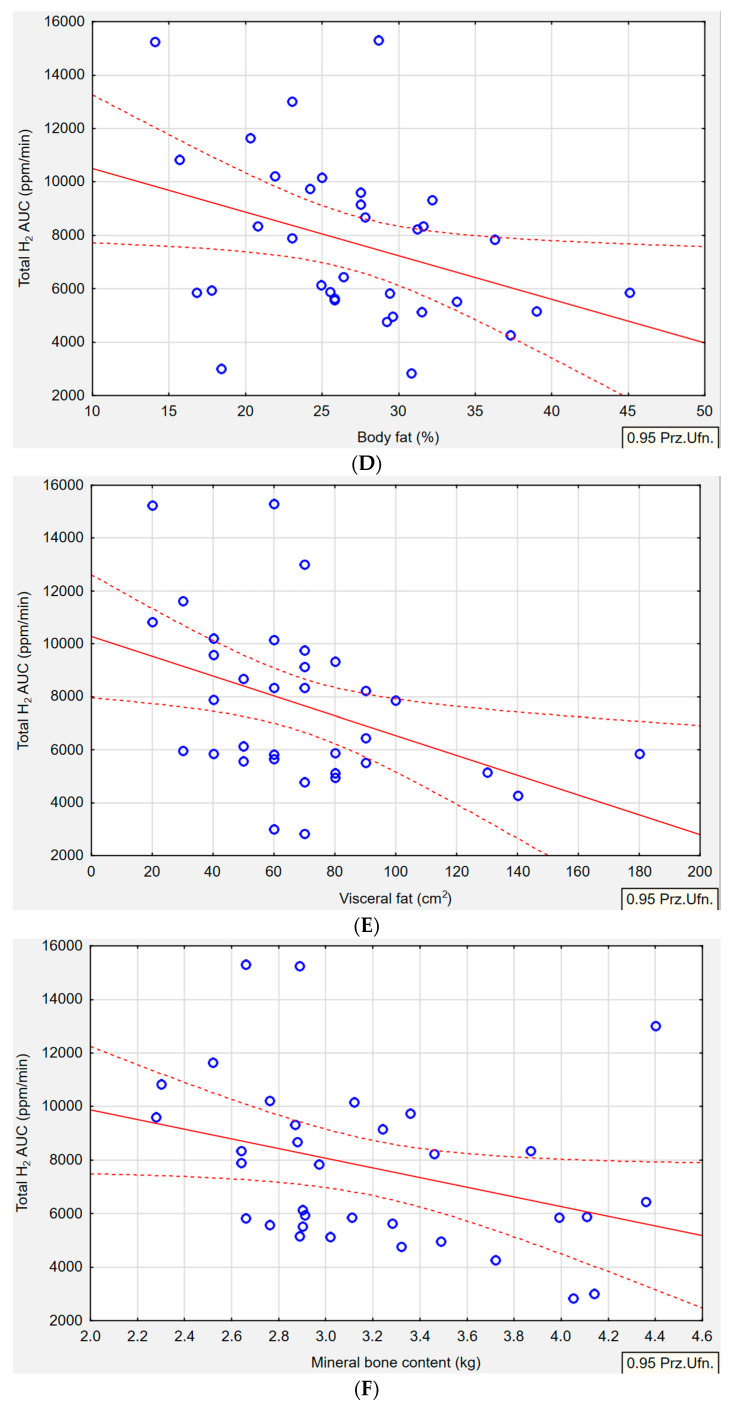

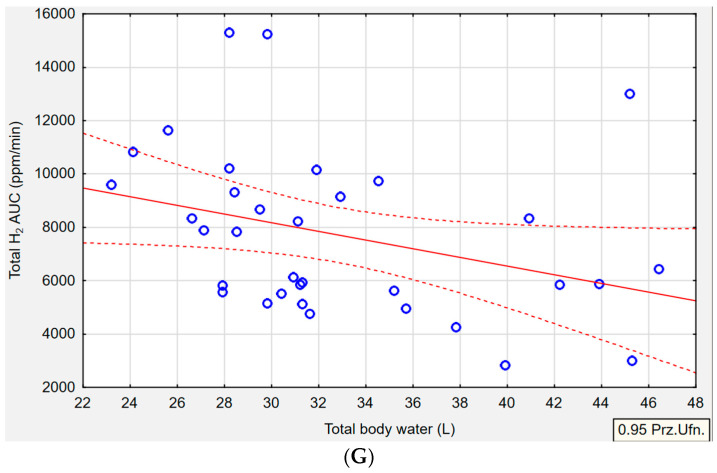
Median H_2_ concentration as area under the curve (ppm/min) in hydrogen–methane-type SIBO by body weight (**A**), BMI (**B**), body fat range (**C**,**D**), visceral fat range (**E**), mineral bone content (**F**), total body water (**G**). Pearson correlation between gas productions and anthropometric measures (*p* ≤ 0.05, r ≥ ±0.3). Red solid line-correlation line, red dotted line-confidence interval, blue circles-cases.

In the H+/M+ group, significant correlations were observed between various anthropometric parameters and H_2_ gas production (Figure 2). Specifically, body weight (*p* = 0.010, r = −0.4308), BMI (*p* = 0.001, r = −0.523), body fat in kg (*p* = 0.009, r = −0.4439), body fat in % (*p* = 0.040, r = −0.3610), visceral fat (*p* = 0.002, r = −0.4035), and mineral bone content (*p* = 0.049, r = −0.3393) showed an inverse association with H_2_ gas production. Additionally, there was a tendency towards an inverse relationship between total body water and H_2_ gas production (*p* = 0.053, r = −0.3344). However, higher concentrations of exhaled CH_4_ were not significantly associated with changes in BMI, body fat (kg or %), WHR, visceral fat, lean body mass, skeletal mass, body protein, or mineral bone content.

## 4. Discussion

In this study, we found a possible link between hydrogen–methane SIBO and selected anthropometric parameters such as BMI, body weight, body fat, visceral fat, mineral bone content, and total body water. To the best of our knowledge, this study represents the first investigation to comprehensively examine a broad range of anthropometric parameters, including muscle mass, skeletal mass, body protein, mineral bone content, and total body water with SIBO type. In previous studies addressing similar research questions, the correlations observed in SIBO patients were mainly related to age, height, BMI, body weight, body fat, and visceral fat [32,33,34,35]. However, in our study, we demonstrated that the high H_2_ gas production in the H+/M+ group was inversely correlated with BMI, body weight, and body fat in kg or in %. Contrary to our results, other authors have noticed a direct association between H+/M+ SIBO and greater BMI and body fat [33]. In our study, neither M+ nor H+ was linked with any anthropometric parameters, regardless of whether we were looking at the concentration of H_2_ or CH_4_ in the exhaled breath. Jung et al. [36] reported a negative association between H+ SIBO and obesity, suggesting that individuals with H+ may exhibit lower BMI. Conversely, Basseri et al. [34] found that CH_4_ production was positively correlated with a higher BMI index among obese individuals. In our article, we observed a negative link between visceral fat and H_2_ levels specifically in the H+/M+ group. However, in a broader population of individuals with SIBO, Fialho et al. [32] reported that an increased visceral to subcutaneous fat ratio was associated with the presence of SIBO. Our study also revealed a noteworthy finding regarding the lower SMM in the H+/M+ group, along with a negative correlation between SMM and H_2_ production. Moreover, another study reported lower bone mineral density in the lumbar spine and femoral neck among individuals with SIBO compared to a reference group [37]. However, when examining the results pertaining to total body water, despite the higher concentrations of exhaled H_2_ gas in the H+/M+ group, we did not observe a higher total body water content. On the contrary, we observed a decrease in total body water, which may be linked with the lower SMM observed in the H+/M+ group.

There are a few possible pathways to explain the observed outcomes, specifically within the H+/M+ group. First of all, the median concentration of exhaled hydrogen gas represented by the AUC during the 180 min breath test in the H+/M+ group (7155 ppm/min) was comparable to the H+ group (6160 ppm/min) but significantly higher than the M+ group (2306 ppm/min). This notable difference in H_2_ concentration may probably account for the correlations observed solely within the H+/M+ group. It is possible that H+/M+ SIBO, characterized by elevated H_2_ gas production, is associated with more profound bacterial fermentation within the small intestine, leading to a greater state of dysbiosis compared to other SIBO subtypes. Gut microbiota dysbiosis and disturbed protective mechanisms of intestinal permeability may be associated with weight loss [38]. Secondly, SIBO has been linked to malabsorption syndromes, resulting in impaired digestion and absorption of various nutrients, ultimately leading to host complications due to alterations in the small bowel microbiota [19,39]. The massive bacterial load in SIBO generates metabolites and toxins that interfere with the intestinal villi’s epithelium, causing disturbances in the activity of small intestinal brush-border disaccharidases and hydrolase enzymes and leading to carbohydrate malabsorption [36,40]. The malabsorption of sugars can result in H_2_ gas production and subsequent diarrhea, leading to underweight status [41]. Poorly digested sugars reaching the large intestine serve as a new source of bacterial colonization, which competes with the host for essential nutrients, negatively impacting the body composition [19]. Thirdly, SIBO contributes to the deconjugation of bile acids in the proximal small intestine [23,39,42]. While bile acids are normally reabsorbed in the ileum, their deconjugation occurs in the jejunum, leading to a magnitude of deficiencies, impaired fat, fat-soluble vitamin absorption, and steatorrhea [6,43]. Deconjugated bile acids irritate and damage the epithelial layer, further promoting protein malabsorption and eventually might deteriorate the bone mass [39,41,42]. Consequently, H_2_ production in H+/M+ may contribute to weight loss, low BMI, decreased body fat mass, visceral fat accumulation, and alterations in total body water, all attributable to impaired absorption of macronutrients and vital vitamins.

The primary limitation of our study is the relatively small sample size, consisting of only 67 participants. The distribution of individuals within each SIBO group is not evenly balanced. Thus, the lack of significant associations for some comparisons in hydrogen SIBO and methane SIBO may be connected to the small sample size. However, it is important to note that our aim was to specifically recruit newly diagnosed SIBO patients in order to minimize the potential influence of treatment therapy on our anthropometric measurements. Another limitation is that the LHMBT is a well available method to diagnose SIBO but it is not the gold standard. Moreover, within the confines of our investigation, individuals afflicted with Small Intestinal Bacterial Overgrowth (SIBO) characterized by elevated hydrogen–sulfide levels were conspicuously omitted from the study, owing to the paucity of accessible diagnostic methodologies in Poland. Nevertheless, this study represents the first attempt to comprehensively evaluate a wide range of anthropometric parameters in SIBO patients. Furthermore, meticulous attention was given to ensure the reliability of both the breath tests and body composition analyses conducted during the examination. Strict adherence to procedures was followed, and qualified professionals with expertise in performing these tests were involved. These rigorous efforts contributed to the robustness and trust worthiness of the data obtained in this study.

## 5. Conclusions

These findings suggest that certain body composition parameters such as body weight, BMI, body fat, and mineral bone content may be inversely correlated with the production of hydrogen in hydrogen–methane SIBO patients. Performing a body composition analysis in SIBO patients should be one of the basic tools to assess and monitor their anthropometric parameters, especially when restrictive diets associated with SIBO treatment can worsen undernutrition. Further research is needed to determine whether the type of SIBO is associated with body composition and specific anthropometric parameters. This will include a larger group of subjects and utilize methane and hydrogen breath tests for diagnosing SIBO.

## Figures and Tables

**Table 1:** Groups characteristics by gas production during the lactulose hydrogen–methane breath.

Gas Production	H+	M+	H+/M+
H_2_	>20 ppm from the baseline within 90 min	<20 ppm from the baseline within 90 min	>20 ppm from the baseline within 90 min
CH_4_	<10 ppm	>10 ppm	>10 ppm

H+—hydrogen-dominant breath, M+—methane-dominant breath, H+/M+—methane–hydrogen breath, H_2_—hydrogen, CH_4_—methane.

**Table 2:** Demographic and clinical characteristics.

	H+ (*n* = 12)	M+ (*n* = 21)	H+/M+ (*n* = 34)	*p*-Value *
Age (years)	35.25 + 11.67	33.29 ± 6.56	32.71 ± 8.23	0.776
*Gender*				0.921
Female	9 (75.0%)	17 (80.9)	27 (79.4%)	
Male	3 (25.0%)	4 (19.1%)	7 (20.5%)	
*Gastrointestinal symptoms*				
Abdominal pain	8 (66.6)	17 (80.9%)	20 (58.8%)	0.242
Diarrhea	9 (75.0%) ^a^	4 (19.0%) ^b^	15 (44.1%) ^ab^	0.007 *
Constipation	3 (25%) ^a^	15 (72.4%) ^b^	18 (52.9%) ^ab^	0.038 *
Reflux	4 (33.3%)	6 (28.5%)	10 (29.4%)	0.957
Gas	8 (66.6%)	16 (76.1%)	31 (91%)	0.118
Growling	7 (58.3%)	12 (57.1%)	28 (82.3%)	0.089
Bloating	10 (83.3%)	20 (95.2%)	32 (94.1%)	0.408
*Frequency of symptoms*				
Once a week	2 (16.6%)	2 (9.5%)	5 (14.7%)	0.934
Few times per week	3 (25.0%)	7 (33.3%)	11 (32.3%)	
Once a day	2 (16.6%)	2 (9.5%)	5 (14.7%)	
Few times per day	5 (41.6%)	10 (47.6%)	13 (38.23%)	

* *p* value is for comparison of differences among the 3 groups, significance level of *p* = 0.05, values calculated with the use of non-parametric Kruskal–Wallis test, verified on the basis of Shapiro–Wilk test; a,b Bonferroni post-hoc test.

**Table 3:** Median hydrogen and methane gas production.

	H+ (*n* = 12)	M+ (*n* = 21)	H+/M+ (*n* = 34)	*p*-Value *
	Median (min-max)	Median (min-max)	Median (min-max)	
*Hydrogen*				
0–90 min AUC (ppm/min)	1260.0 (580.0; 2335.0) ^a^	310.0 (0.0; 2400.0) ^b^	1172.5 (405.0; 4455.0) ^a^	0.001
90–180 min AUC (ppm/min)	4705.0 (2660; 8225.0) ^a^	2045.0 (40.0; 10,425.0) ^b^	5322.5 (1960.0; 11,315.0) ^a^	0.001
Total AUC (ppm/min)	6160.0 (3630.0; 10,560.0) ^a^	2360.0 (140.0; 12,825.0) ^b^	7155.0 (2848.0; 15,310.0) ^a^	0.001
*Methane*				
Basal (ppm)	0.0 (0.0; 3.0) ^a^	15.0 (3.0; 54.0) ^b^	6.5 (0.0; 27.00) ^c^	0.001
Total AUC (ppm/min)	532.5 (10.0; 930.0) _a_	4510.0 (1340.0; 13,090.0) ^b^	2267.5 (1060; 9850.0) ^b^	0.001

* *p* value is for comparison of differences among the 3 groups, significance level of *p* = 0.05, values calculated with the use of non-parametric Kruskal–Wallis test, verified on the basis of, Shapiro–Wilk test, a–c Bonferroni post-hoc test.

**Table 4:** Comparisons between the SIBO types with anthropometric parameters based on bioimpedance test.

	H+ (*n* = 12)	M+ (*n* = 21)	H+/M+ (*n* = 34)	*p*-Value *
	Median (min-max)	Median (min-max)	Median (min-max)	
Height (cm)	172.5 (158.0–192.0) ^a^	170.0 (159.0–185.0) ^ab^	166.0 (153.0–189.0) ^b^	0.023 *
Weight (kg)	65.7 (45.0–109.8)	62.2 (45.8–92.3)	61.3 (39.0–86.2)	0.236 ^+^
Body fat (%)	29.9 (17.3–36.0)	24.4 (11.4–46.4)	26.9 (14.1–45.1)	0.924 ^+^
Body fat (kg)	19.4 (10.9–37.3)	13.7 (6.5–42.6)	16.35 (6.1–35.1)	0.147 ^+^
Lean Body mass (kg)	47.5 (34.1–81.1)	48.4 (37.8–64.6)	42.25 (31.7–63.4)	0.104 ^+^
Skeletal muscle mass (kg)	26.0 (18.1–46.0) ^a^	26.8 (20.4–36.8) ^a^	23.1 (16.6–36.0)^b^	0.044 ^+^
Total Water (L)	31.2 (23.9–59.4)	35.0 (26.5–47.1)	31.1 (23.2–46.4)	0.396 *
Body protein (kg)	8.3 (6.4–15.9)	9.5 (7.4–13.9)	8.4 (6.2–12.6)	0.206 ^+^
Mineral (kg)	3.3 (2.4–5.8)	3.4 (2.7–4.6)	2.9 (2.3–4.4)	0.392 ^+^
Visceral fat (cm^2^)	80 (40.0–170.0)	50.0 (20.0–190.0)	65.0 (20.0–180.0)	0.170 ^+^
BMI (kg/m^2^)	21.8 (18.0–32.3)	21.0 (17.2–32.6)	21.7 (16.7–27.6)	0.711 ^+^
WHR	0.87 (0.74–1.1)	0.86 (0.74–0.99)	0.85 (0.76–1.0)	0.715 *

*p* value is for comparison of differences among the 3 groups, significance level of *p* = 0.05; *—values calculated with the use of non-parametric Kruskal–Wallis test, verified on the basis of Shapiro–Wilk test, a,b Bonferroni post-hoc test; +—values calculated with the use of parametric Tukey test.

**Table 5:** The percentage of patients in three groups in the indicated classifications of BMI, body fat and visceral fat area.

	Classification									
	BMI				Body Fat			Visceral Fat Area		
	underweight	healthy	overweight	obese	underfat	normal	overfat	standard	high	very high
H+	8%	67%	17%	8%	9%	38%	53%	88%	9%	3%
M+	13%	67%	10%	10%	14%	57%	28%	86%	5%	9%
H+/M+	18%	64%	18%	0%	0%	34%	66%	75%	17%	8%
*p*-Value *	*p* = 0.991				*p* = 0.597			*p* = 0.548		

* *p* value is for comparison of differences among the 3 groups, significance level of *p* = 0.05, values calculated with the use of non-parametric Kruskal–Wallis test, verified on the basis of Shapiro–Wilk test.

## Data Availability

The data presented in this study are available on request from the corresponding author.
